# Supplementary material for: Association between oral microbiome and seven types of cancers in East Asian population: a two-sample Mendelian randomization analysis
Source: Front Mol Biosci. 2023 Nov 21;10:1327893. doi: 10.3389/fmolb.2023.1327893 (PMC10702768; doi:10.3389/fmolb.2023.1327893)
Supplement: Supplementary file 1 [file DataSheet1.ZIP › Supplementary Materials/MR plots of saliva/saliva═╝/Pancreatic cancer/pheno.1375_to_pancreatic cancer_forest.pdf]

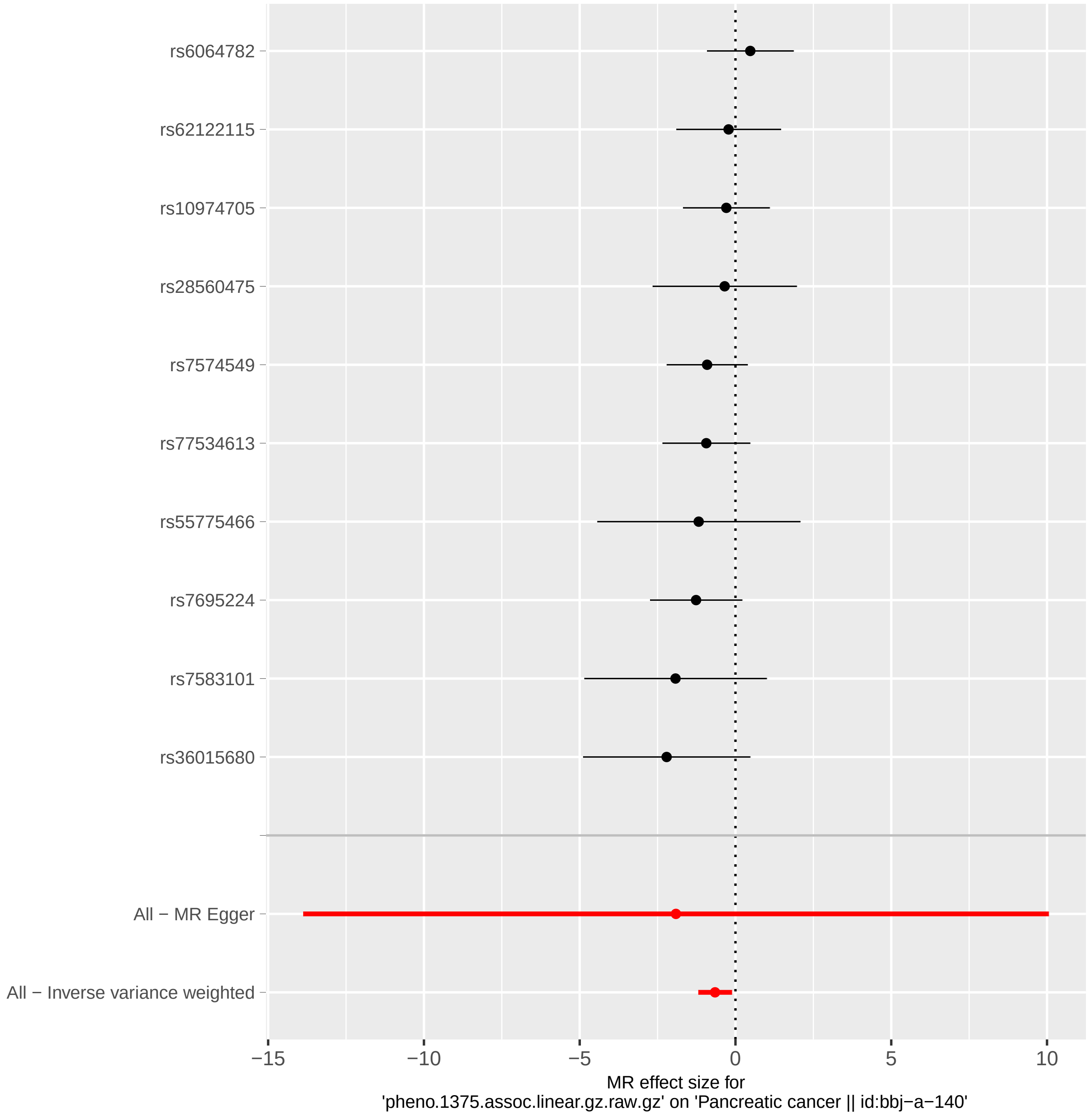
rs6064782
rs62122115
rs10974705
rs28560475
rs7574549
rs77534613
rs55775466
rs7695224
rs7583101
rs36015680
All − MR Egger
All − Inverse variance weighted
−15
−10
−5
0
5
10
MR effect size for
'pheno.1375.assoc.linear.gz.raw.gz' on 'Pancreatic cancer || id:bbj−a−140'
